# Putative Genomic Signatures of Local Adaptation in Five Local Indonesian Sheep Reveal Selection on Immunity, Reproduction, and Production Traits

**DOI:** 10.1002/ece3.73665

**Published:** 2026-06-01

**Authors:** Putri Kusuma Astuti, Alexandru Eugeniu Mizeranschi, Herdis Herdis, Pradita Iustitia Sitaresmi, Alek Ibrahim, Sigit Prastowo, Nuzul Widyas, Tristianto Nugroho, Szilvia Kusza

**Affiliations:** ^1^ Centre for Agricultural Genomics and Biotechnology, Faculty of Agricultural and Food Sciences and Environmental Management University of Debrecen Debrecen Hungary; ^2^ Research and Development Station for Bovine Arad Romania; ^3^ Institute for Advanced Environmental Research West University of Timisoara Timisoara Romania; ^4^ Research Center for Animal Husbandry National Research and Innovation Agency, Cibinong Science Center Bogor Indonesia; ^5^ Department of Animal Science, Faculty of Animal Science Universitas Sebelas Maret Surakarta Indonesia; ^6^ Department of Animal Production, Faculty of Animal Science Universitas Gadjah Mada Yogyakarta Indonesia

**Keywords:** Indonesia, local breed, *Ovis aries*, population structure, putative signature of selection

## Abstract

Indonesian sheep, shaped by survival, reproduction and productivity in humid tropical climates, may carry genomic regions associated with local adaptation and economically important traits that remain largely unexplored. This study performed genetic diversity analysis on 17,534 SNPs derived from the OvineSNP50 BeadChip genotyping array, involving 120 individuals among five local Indonesian sheep breeds: Batur, Garut, Sakub, Sumatra and Thin‐tail. Analysis of genetic structure, incorporating PCA, maximum‐likelihood phylogenetics analysis and Admixture analyses, demonstrated that each population displayed a uniquely homogeneous and distinctive ancestry profile, with the exception of Sakub and Thin‐tail. Additionally, a close genetic relationship among Sakub, Garut and Thin‐tail sheep was identified. We employed four complementary approaches of within and cross population selection signature analyses to identify putative selection signatures within 13 genomic regions. Several genes identified within the selection signature regions are potentially associated with the immune system (e.g., *ATRNL1*, *DDX58* and *NELFCD*), wool traits and pigmentation (e.g., *CERS4* and *EDN3*), reproductive traits (e.g., *GNPDA2, NAMPT* and *NDRG1*), milk traits (e.g., *SLC39A8, ST3GAL1* and *EVI5L*), meat traits (e.g., *PIK3CG, CDHR3* and *ANGPTL4*), and adaptive traits (e.g., *SLC26A4* and *NDUFB6*). Understanding the candidate genomic areas under selective pressure in sheep breeds may aid in identifying the associated genes and improve our comprehension of their involvement in local adaptation. Consequently, this research provides a preliminary genomic resource for the conservation and utilization of these sheep genetic resources in Indonesia.

## Graphical abstract

We determined the genetic structure and signatures of selection in five local Indonesian sheep breeds using genome‐wide SNP data. By integrating population structure analyses with runs of homozygosity and haplotype‐based selection scans, we identified breed‐specific genomic regions associated with immunity, reproduction, pigmentation, metabolism, and environmental adaptation. Our findings provide new insights into the evolutionary processes shaping locally adapted livestock populations in tropical environments.

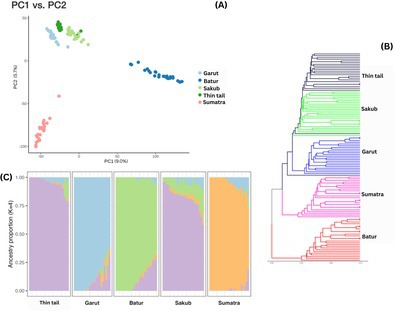

## 1 Introduction

Genomic approaches such as the SNP chip array have been proven to be able to effectively do genetic screening to pinpoint particular areas of the genome and potential genes that contribute to the specific traits of various sheep breeds. Unfortunately, this advanced technique remains limited in many Asian countries and developing nations owing to a lack of financial and infrastructural resources, while animals from these countries might have a great potential in terms of local adaptation and production that are still left unexplored. For example, while genomic scans using the OvineSNP50 BeadChip have been conducted in several parts of Asia (Saravanan et al. 2020; Dotsev et al. 2025; Yousefi et al. 2025; Eydivandi et al. 2021), the overall number of studies remains comparatively limited when viewed at a global scale, with the exception of areas in inner Asia (Deniskova et al. 2019; Pozharskiy et al. 2020) where sheep studies are at an advanced stage. This gap becomes particularly evident in Southeast Asia, where native sheep populations have received minimal genomic attention and remain largely unexplored. Similar to most developing nations, in Indonesia, sheep are mostly kept by smallholder farmers due to their low cost and easy maintenance, and they are mostly located in areas with limited resources. Despite their role in nutrition resource (mainly as meat producer), sheep also important for Indonesian religious and cultural activities (Budisatria et al. 2009). As per 2024 statistic report (https://www.bps.go.id), the Indonesian sheep population was accounted for 9.219.176 heads, with around 75.5% of them found in West Java. As many as 10 local sheep breeds have been registered by the ministry of Agriculture of Indonesia, while several others are still not officially recognized, some of them are Batur, Sakub, Thin‐tail, Garut and Sumatra.

Originating in the Batur District of Banjarnegara Regency in Central Java, Indonesia, Batur sheep are a hybrid breed of Merino and Thin‐tail sheep, which has been developed since 1974. This sheep is commonly found in the mountainous regions with exceptional adaptability to the cold and humid climate (18°C–21°C) (Ibrahim, Budisatria, Widayanti, and Artama 2020). Mainly found in Brebes Regency, the Sakub sheep are a popular meat breed with a lineage that includes Texel, Suffolk, Merino, and indigenous sheep; this breed has only just gained formal recognition in 2022 (Nurasih et al. 2023). The Thin‐tail sheep is the indigenous breed predominantly maintained by local farmers to this day. The Garut sheep is mostly raised in West Java, with a history of crossbreeding indigenous thin‐tailed sheep with Australian Merino, Fat‐tail, and Kaapstad sheep from Africa (Udo and Budisatria 2011; Ibrahim, Budisatria, Widayanti, and Artama 2020). The Sumatran sheep is one of the Thin‐tailed sheep breeds with coarse wool. The origin of the Sumatran sheep is not yet known for certain but could be linked to the Javanese Thin‐tail (Priyanto et al. 2000).

The all‐time main issue of Indonesian livestock biodiversity is the common occurrence of indiscriminate crossbreeding and insufficient genetic monitoring programs (Hastarina et al. 2025), causing untraceable and uncontrollable genetic admixture (Widyas et al. 2022), which can also be easily noticed from the unclear breed history of the previously mentioned breeds. The study on genetic characterization of these valuable indigenous livestock is also still lacking; meanwhile, it is emphasized by Hastarina et al. (2025) that selective breeding strategies to increase resilience, production, and adaptation can be optimized by integrating genomics data with phenotypic and ecological information.

This study, to the best of our knowledge, would be the first study in genomic characterization of Indonesian local sheep breeds. The objective of this study is to reveal breed‐specific putative selection signatures of these genomically unexplored sheep breeds of Indonesia using the medium density Ovine50K SNPs array, together with the possibility to clarify their genetic composition. The findings provide preliminary candidate regions and genes of interest that may support future selective breeding and conservation management programs.

## 2 Materials and Methods

### 2.1 Sample Collection and Genotyping

**FIGURE 1:**
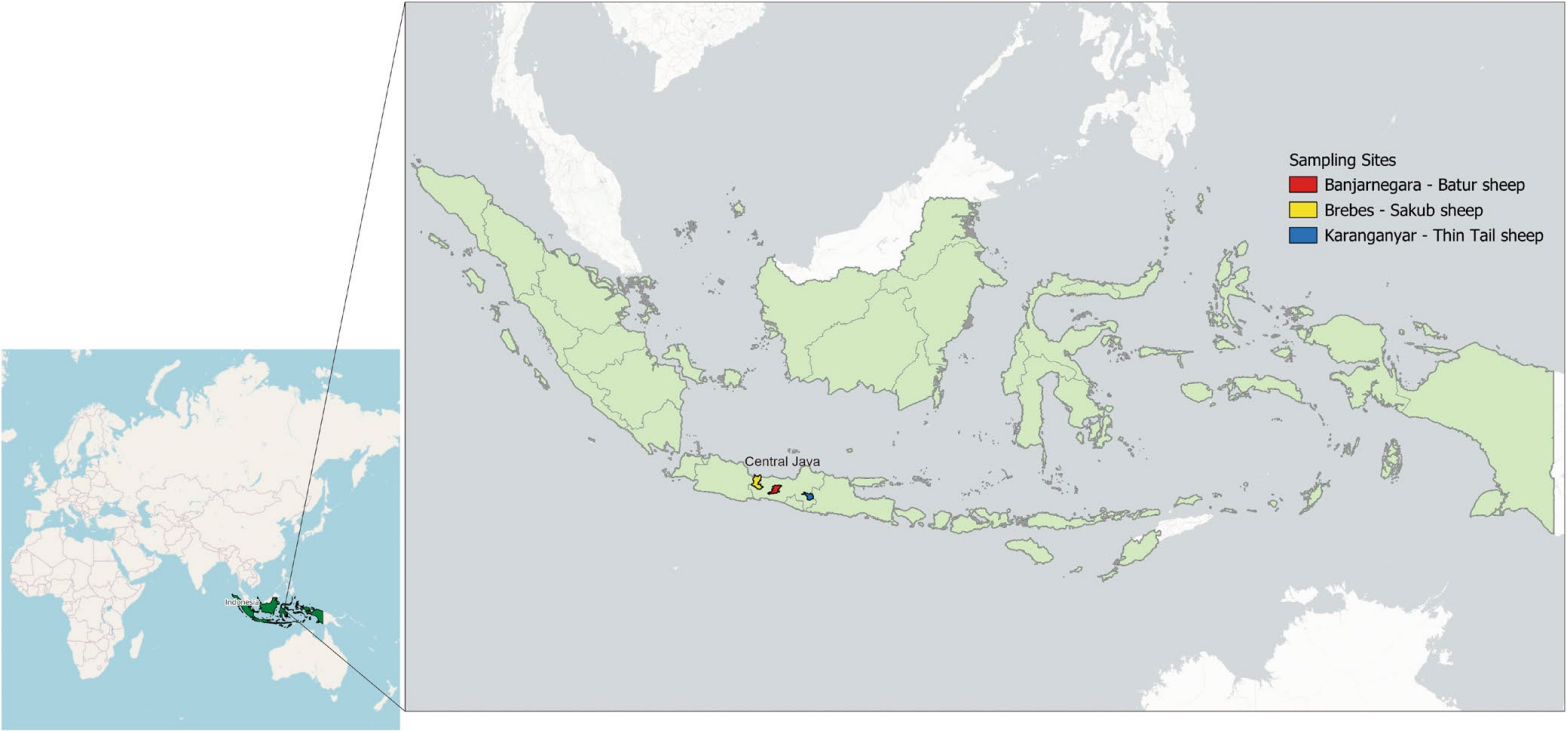
Sampling site for the 75 animals that were freshly genotyped for this study, i.e., Batur (*n* = 26), Sakub (*n* = 25), and Thin‐tail (*n* = 24).

In the present study, 75 blood samples of three Indonesian indigenous sheep breeds (25 Sakub, 26 Batur and 24 Thin‐tail) from three regions in Central Java, Indonesia (Figure 1) were freshly genotyped. Additionally, data for two sheep breeds: Garut (*n* = 22) and Sumatra (*n* = 24) were obtained from the publicly accessible Sheep HapMap database (https://sheephapmap.org).

Blood samples were collected from the jugular vein of the sheep. Genomic DNA was extracted and isolated from blood of Sakub and Batur sheep using the DNeasy blood and tissue kit (Qiagen, Germany), following the manufacturer's protocol and subsequently sent to Neogen in Scotland for genotyping, which was performed using the Ovine SNP50K Chip (Illumina, USA). However, blood samples from Thin‐tail sheep were collected and directly transferred onto FTA cards (Neogen, USA) for transport. The whole pipeline from DNA isolation to variant call was performed by the genotyping company, resulting in 47,180 SNPs with a genotyping rate of 0.99.

### 2.2 Bioinformatics Analysis

Genotypes were converted to the sheep reference genome version ARS‐UI_Ramb_v2.0 using the LiftoverVcf command from Picard, using the GATK package v4.6.1.0 (Van Der Auwera and O'Connor 2020). Merging was performed with PLINK v1.9 (Purcell et al. 2007) and bcftools v1.21 (Li 2011). Quality control was performed with PLINK v1.9 (Purcell et al. 2007) using the options “‐‐geno 0.05 ‐‐mind 0.05 ‐‐maf 0.05”, followed by minor allele frequency (MAF), observed heterozygosity (Ho), expected heterozygosity (He), and inbreeding coefficient (F) calculation.

Population structure was assessed using both fastStructure v1.0 (Raj et al. 2014) and Admixture v1.3.0 (Alexander et al. 2009) with a prior process of linkage disequilibrium (LD) pruning using ‐‐indep‐pairwise 50 5 0.2. Principal component analysis was performed with the argyle package v0.2.2 (Morgan 2015). Phylogenetic analysis was performed using the R packages ape v.5.8‐1 (Paradis and Schliep 2018) and phangorn v2.12.1 (Schliep 2010) in RStudio 2026.01.1 + 403 (Posit Team 2026). The function phangorn::modelTest was used to test all the available genetic substitution models. The optimal model was then used to infer an ultrametric phylogenetic tree using the phangorn::pml_bb function with the options *k* = 0 and rearrangement = “NNI”. The resulting tree was visualized via the R package tidytree (Yu 2022). Admixture v1.3.0 (Alexander et al. 2009) was used for population ancestry examination with K being set to 2–10 and the cross‐validation (CV) error was recorded per each *K*. The optimal *K* was identified as the one with the lowest CV error and resulting *Q*‐matrix files containing individual ancestry coefficients were visualized using the tidyverse (Yu 2022) and ggplot2 (Wickham 2016) packages.

For the detection of within‐population putative selection signatures, unpruned data per breed was used, which was phased and imputed using Beagle v5.2 (Browning et al. 2021). Runs of homozygosity (ROH) and the genomic inbreeding coefficient (*F*_ROH_) were obtained with detectRUNS (Biscarini et al. 2018) using the sliding‐window method with: window size = 15 SNPs; SNP‐state threshold = 0.05; minSNP per run = 3; maxOppWindow = 1; maxMissWindow = 1; minLength = 1 Mb; minDensity ≥ 1 SNP/100 kb. ROH were binned into five length classes (1–6, 6–12, 12–24, 24–48, > 48 Mb). ROH islands were defined as consecutive SNPs (no gap) where SNPs are inside runs in more than 60% of the individuals in the population. Additionally, the Integrated Haplotype Score (iHS) approach was employed to identify signature of selection using the Rehh package (Gautier et al. 2016). Candidate regions were determined and identified by a sliding window approach: non‐overlapping centers with 500‐kb windows and 250‐kb step; windows with ≥ 5 SNPs were retained and candidate regions were defined as windows in the top 1% of the empirical distribution of mean |iHS| within each breed.

For cross population analysis Pairwise $F_{\mathrm{ST}}$ and XP‐EHH selection scans were performed. Pairwise $F_{\mathrm{ST}}$ was calculated for all 10 breed combinations with a window size of 500 kb and a step size of 250 kb with at least three SNPs. The XP‐EHH was done using the rehh package (Gautier et al. 2016) and candidate regions were defined from the top 1% of the empirical distribution of absolute XP‐EHH values.

Results from pairwise $F_{\mathrm{ST}}$ and XP‐EHH were interpreted together with the within‐population ROH island and iHS analyses. Candidate intervals from ROH, iHS, Pairwise $F_{\mathrm{ST}}$, and XP‐EHH were converted to a common genomic interval using a maximum gap of 250 kb. Regions supported by multiple complementary methods were prioritized as the strongest putative selection signatures. Some other regions showed support from two complementary methods, where a within‐population signal (ROH or iHS) coincided with a between‐population signal (FST or XP‐EHH) involving the same breed, were retained as secondary putative selection signatures. Visualization was done using *ggplot2 package* (Wickham 2016).

Candidate regions were annotated locally against the ARS‐UI_Ramb_v2.0 sheep reference using the assembly‐specific GFF file in R. These genes were subsequently subjected to gene ontology (GO) and Kyoto Encyclopedia of Genes and Genomes (KEGG) pathway analysis using the DAVID online database (https://davidbioinformatics.nih.gov) with a nominal significance threshold of *p* < 0.05.

## 3 Results

After quality control, one Batur sheep sample did not pass the filtering due to missing genotype data and 17,534 SNPs from all five sheep breeds (*n* = 120) remained for downstream analyses, with a total genotyping rate of 0.998. This significant reduction in variant numbers is mainly due to the different reference genome of the both sources of data (own genotyping and HapMap public data), which were converted to ARS‐UI_Ramb_v2.0.

The five Indonesian sheep breeds exhibited comparable levels of genetic diversity, as indicated by similar MAF, Ho and He values (Table 1). Garut sheep showed the highest observed heterozygosity (He = 0.349), while Batur had the highest MAF (MAF = 0.275). Inbreeding coefficients were relatively low in all breeds (−0.038 < *F* < 0.025).

**TABLE 1:** Population genomics parameters of the five Indonesian sheep breeds.

Breed	*N*	MAF	Ho	He	F
Batur	25	0.275	0.336	0.324	−0.038
Garut	22	0.259	0.349	0.339	−0.029
Sakub	25	0.259	0.346	0.344	−0.007
Sumatra	24	0.261	0.323	0.328	0.017
Thin tail	24	0.247	0.326	0.335	0.025

Abbreviations: F, inbreeding coefficient; He, Expected heterozygosity; Ho, Observed heterozygosity; MAF, Minor allele frequency; *N*, number of sample.

**FIGURE 2:**
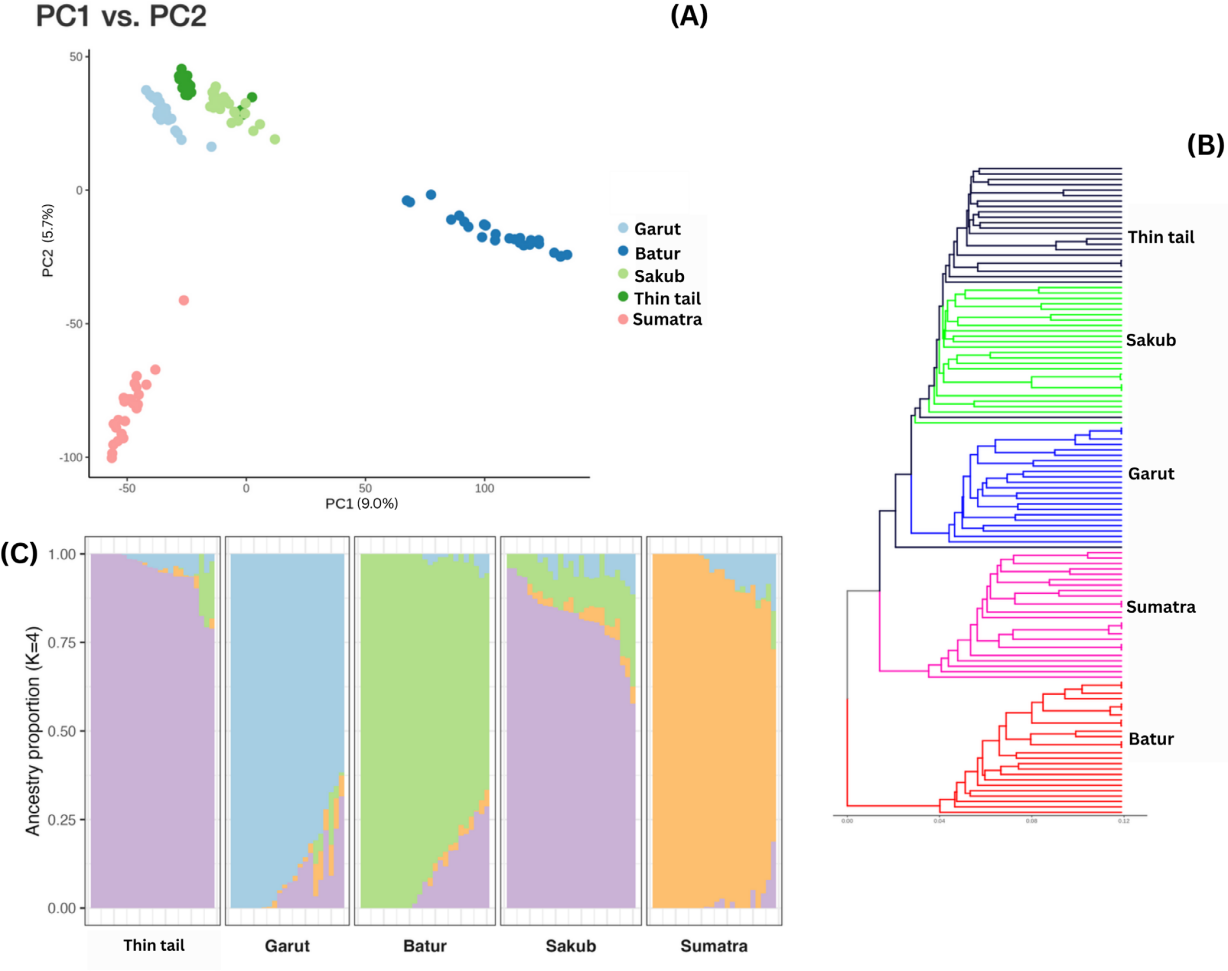
Population structure analyses: (A) PCA, (B) Phylogenetics tree and (C) Admixture analysis at *K* = 4 of the 5 Indonesian local sheep breeds; Batur, Garut, Sakub, Sumatra and Thin‐tail sheep.

To assess genetic structure and relationships among populations using PCA, *F*_ST_ and Admixture analyses, LD pruning was performed, resulting in 16,665 SNPs. The PCA results demonstrated significant relationships among the Garut, Sakub and Thin‐tail breeds (Figure 2A), supported by their clustering on the same side of the branch in the phylogenetic tree depicted in Figure 2B. Separate clades in the phylogenetic tree indicated that Batur and the other four sheep breeds (Garut, Sakub, Thin‐tail and Sumatra) shared a different ancestor. The first and second principal component (PC1 and PC2, Table S1), accounting for 9.0% and 5.7% of the total variation, respectively, of the total genetic variance among these five breeds, distinctly divides these three breeds from the other two (Sumatra and Batur), hence creating three separate clusters. Overlapping Sakub and Thin‐tail individuals suggest a close relationship or admixture between the two breeds. The Admixture analysis to reconstruct population structure and ancestry mixing showed the optimal *K* value was *K* = 4 (Table S2) that best explained the population structure based on the lowest CV error (0.548), where each breed primarily and consistently constituted its own ancestral component. Each population demonstrated a notably homogeneous and specific ancestry profile (> 54% breed‐specific ancestry), except for Sakub and Thin‐tail that seem to share the same ancestry, colored in purple as shown in Figure 2C.

### 3.1 ROH Distribution and Genomic Inbreeding

With the sliding window methodology, we discovered a total of 14,598 ROH segments among five sheep populations (Table 2). Of all identified ROHs, 12,800 were between 1 and 6 Mb, 1108 ranged from 6 to 12 Mb, 507 from 12 to 24 Mb, 155 from 24 to 48 Mb, and 19 segments over 48 Mb (Table 2). In this study, across all breeds, we observed a higher number of short ROH segments (< 6 Mb) and a lower proportion of long ROHs (> 48 Mb). In the short length category (1–6 Mb), the Thin tail breed had the highest count of ROHs (3106), however, in the long length category (> 48 Mb), the Batur breed demonstrated the highest count (6 ROHs). The allocation of the total count of ROHs per chromosome and the percentage of chromosomes exhibiting ROH in each sheep breed is detailed in Table S3. The biggest number of ROH was recorded in Sumatra sheep (*n* = 191), whereas the smallest was noted in Batur sheep (*n* = 74). The mean genomic inbreeding coefficient derived from runs of homozygosity (*F*_ROH_) was highest in Sumatra (0.250) and lowest in Sakub (0.146) (Table S3).

**TABLE 2:** Count of runs of homozygosity per each of the five Indonesian sheep breeds and length class.

Class (Mb)	Batur		Garut		Sakub		Sumatra		Thin tail	
	*N*	%	*N*	%	*N*	%	*N*	%	*N*	%
1–6	1820	74.99	2415	90.86	2555	93.73	2904	84.84	3106	92.58
6–12	324	13.35	166	6.25	122	4.48	354	10.34	142	4.23
12–24	217	8.94	62	2.33	34	1.25	127	3.71	67	2.00
24–48	60	2.47	11	0.41	14	0.51	35	1.02	35	1.04
> 48	6	0.25	4	0.15	1	0.04	3	0.09	5	0.15

### 3.2 Signature of Selections

**FIGURE 3:**
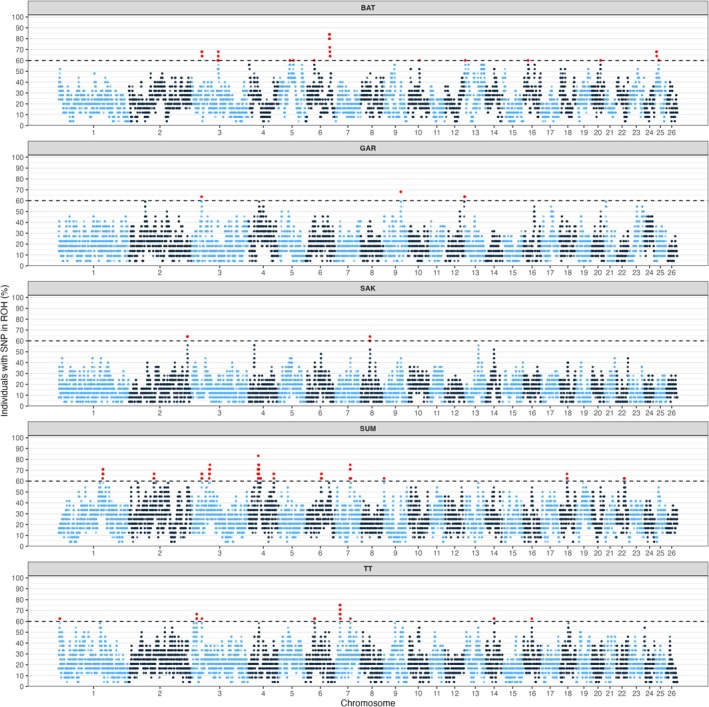
Manhattan plot of the distribution of SNPs in ROH islands across the chromosomes in five Indonesian sheep breeds: Batur (BAT), Garut (GAR), Sakub (SAK), Sumatra (SUM), and Thin‐tail (TT). *X*‐axis shows the distribution of ROHs and *Y*‐axis represents the frequency (%) of overlapping ROH shared among the individuals.

**FIGURE 4:**
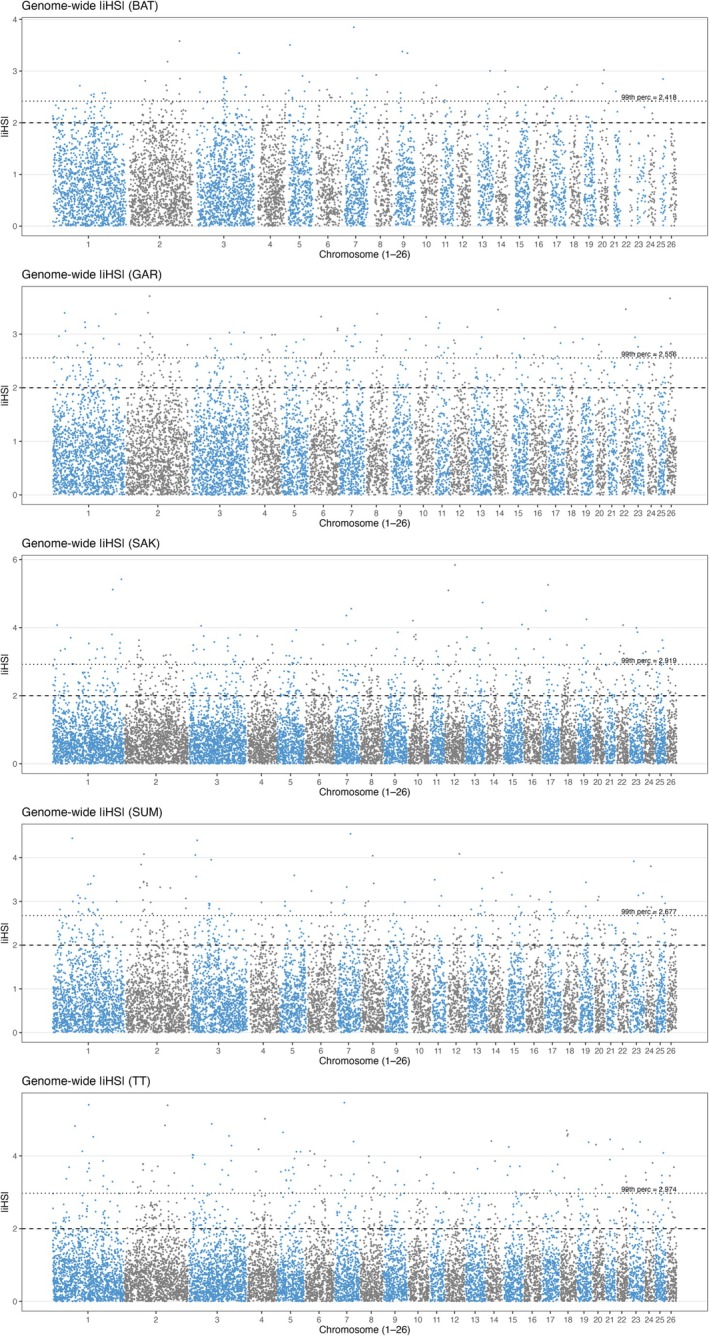
Genome‐wide distribution of standardized iHS values in the five Indonesian local sheep breeds: Batur (BAT), Garut (GAR), Sakub (SAK), Sumatra (SUM), and Thin‐tail (TT). The horizontal line shows the cut‐off iHS with top 1% value to call SNP outliers.

**FIGURE 5:**
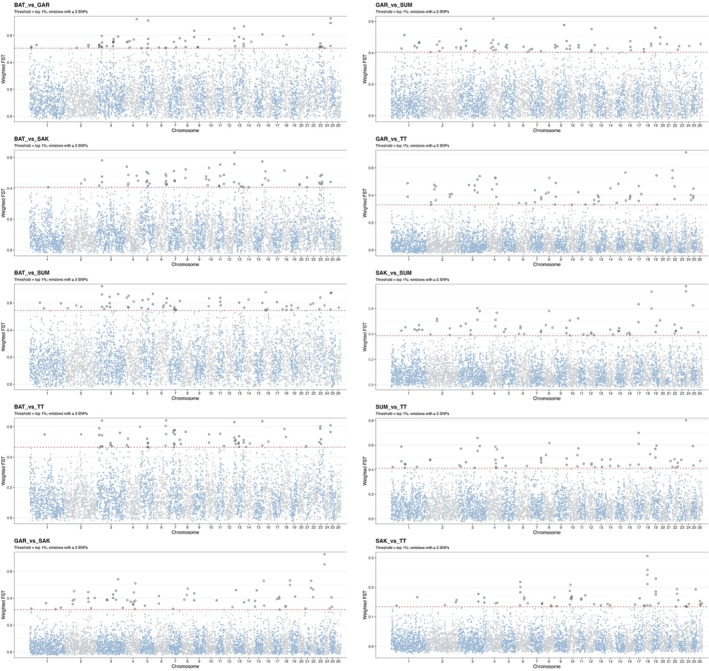
Genome‐wide distribution of Pairwise Fst values with a window size of 500 kb and a step size of 250 kb with at least three SNPs in the 5 Indonesian local sheep breeds: Batur (BAT), Garut (GAR), Sakub (SAK), Sumatra (SUM), and Thin‐tail (TT).

**FIGURE 6:**
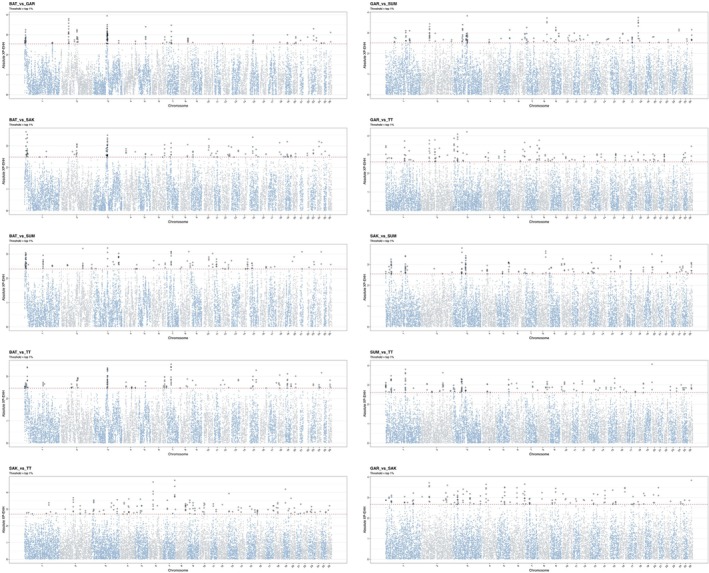
Genome‐wide distribution of standardized XP EHH values in the 5 Indonesian local sheep breeds: Batur (BAT), Garut (GAR), Sakub (SAK), Sumatra (SUM), and Thin‐tail (TT). The horizontal line shows the cut‐off XP EHH with top 1% value to call SNP outliers.

Integration of within‐ (Figures 3 and 4) and between‐population (Figures 5 and 6) analyses highlighted several putative candidate regions (Tables S4–S7). The robust putative selection signatures detected in a broad region on chr 3 (40.50–43.24 Mb) were supported by ROH and cross‐population analyses and represented a Batur/Sumatra‐associated hotspot; this was neglected due to unspecified single population. Three regions were consistently centered on Sumatra, including chr 3 (70.56–74.82 Mb), chr 4 (47.99–50.23 Mb), and chr 18 (60.03–60.78 Mb), each supported by both within‐ and between‐population evidence.

Some other regions showed support from two complementary methods were retained as secondary putative selection signatures. For Sumatra sheep, includes chr 2 (99.88–101.75 Mb), chr 6 (62.08–64.53 Mb), chr 22 (33.62–35.38 Mb), and chr 1 (155.96–157.84 Mb). For Batur sheep, include chr 6 (22.73–23.23 Mb) and chr 2 (86.65–87.46 Mb). For Sakub sheep, include chr 2 (162.53–163.53 Mb) and chr 9 (20.34–21.43 Mb), while for Thin tail sheep includes chr 5 (14.26–14.96 Mb) and chr 13 (56.70–57.20 Mb).

### 3.3 Gene Ontology and Pathway Analysis

A total of 141 genes were identified within 13 selected regions (Tables S8 and S9) across the Indonesian sheep breeds, with no candidate region detected in Garut sheep. These genes were subjected to GO and KEGG enrichment analysis in DAVID using a nominal significance threshold of *p* < 0.05. Several GO categories showed nominal enrichment (Table S10); however, no GO terms or KEGG pathways remained significant after multiple‐testing correction. Therefore, these enrichment results should be considered exploratory only.

## 4 Discussion

Although minor differences were observed, the overall pattern suggests that these breeds retain relatively similar and moderate within‐population genetic diversity. Population‐structure analyses based on PCA, phylogenetic analysis and Admixture were conducted to reveal the distinct genetic clusters reflecting both ecological adaptation and historical patterns of gene flow. According to the phylogenetics tree and PCA results, Sakub, Garut, and Thin‐tail breeds share a close genetic relationship due to their near proximity to one another. Similar findings from an earlier study using RAPD markers (Prayitno et al. 2011) indicated that the Garut and Thin‐tail share a strong genetic relationship. Sakub and Thin‐tail sheep shared a common ancestry according to Admixture analysis, which was also found in Puspita et al. (2025) that these two breeds are closely related based on the MtDNA d‐loop sequence. The ancestry pattern of Sumatra sheep is different with the Thin‐tail sheep, supporting the idea of Tiesnamurti et al. (2021), that the Sumatra sheep could be related to Indian sheep which were brought by traders during the 19th century, not the Thin‐tail sheep. This Admixture result has shown a small amount of thin‐tail specific ancestry in every breed, which lends validity to the idea that traditional farms often engage in uncontrolled cross‐breeding with this particular sheep breed, as the most common breed to be kept by local farmers.

The genomic distribution, length, and frequency of ROH can be used to determine the demographic history of livestock. The degree of relatedness and inbreeding across breeds is shown by the length of consecutive homozygous segments. Ping et al. (2025) suggested that a short ROH suggests a larger population with more distant common ancestors, whereas the long ROHs are primarily linked to recent inbreeding. Among the five breeds, Batur sheep were found to have the highest proportion of long ROH (2.47% of 24–48 MB and 0.25% of > 48 MB), suggesting a higher recent inbreeding level compared to other breeds, supporting Ibrahim, Budisatria, Widayanti, Atmoko, et al. (2020)'s finding regarding detection of inbreeding sign due to significant population decline of Batur sheep in recent years. However, the average genomics F_ROH_ showed that Sumatra sheep (0.250) have the highest inbreeding level, followed by Batur sheep (0.225). This finding is somewhat concerning as these values far exceed those recorded in Iranian sheep (Afshari and Qezel, both at 0.005) and in Turkish breed (Karakas, Norduz, and Sakiz, ≤ 0.15; Bayraktar 2024; Yousefi et al. 2025).

Multimethod detection of putative genes under selection was employed to improve robustness against confounding from population structure and residual admixture, with ROH and iHS performed within breeds and only regions consistently supported across ROH, iHS, pairwise Fst and XP‐EHH prioritized as the most reliable putative selection signatures. The set of putative genes identified in this study (*n* = 141) comprised 5 genes for Batur, 11 for Sakub, 80 for Sumatra and 45 for Thin‐tail sheep and may reflect breed‐specific selection signals that warrant further validation (Table 3). Some of these genes have previously been reported in association with production, reproduction, immunity or adaptation‐related traits in sheep. This number of breed‐specific positive selection of signature is relevant in comparison to reported work in Hu sheep (*n* = 206) with the same approach on whole genome sequencing data (Zhao, Xie, et al. 2024) and less than what was reported in Iraqi Awassi (*n* = 259) and Hamdani (*n* = 563) sheep (Bayraktar et al. 2025). It is important to point out that we integrated the process of converting data from two distinct sources (own data and HapMap consortium data) to a unified reference genome (ARS‐UI_Ramb_v2.0), resulting in only approximately 32% of our genotyping data being considered suitable for the subsequent analysis. This step was done to ensure precise alignment of variants across samples, thereby preventing silent mismatches and incorrect conclusions.

**TABLE 3:** Selection signatures detected by multimethod analyses (ROH, iHS, Pairwise Fst and XP‐EHH) in five Indonesian sheep breeds.

Breed	Chr	Region span (Mb)	Gene names	Analyses supported
Sumatra	3	70.56–74.82	*LOC114114263*; *TRNAC‐GCA*; *NRXN1*; *TRNAS‐GGA*; *TRNAW‐CCA*	ROH + iHS + XP‐EHH
	4	47.99–50.23	*SRPK2*; *LOC114114604*; *PUS7*; *RINT1*; *EFCAB10*; *ATXN7L1*; *TRNAG‐UCC*; *LOC114114651*; *CDHR3*; *LOC105609617*; *LOC121819397*; *SYPL1*; *LOC114114678*; *NAMPT*; *CCDC71L*; *PIK3CG*; *PRKAR2B*; *LOC121819398*; *HBP1*; *COG5*; *GPR22*; *DUS4L*; *BCAP29*; *LOC114114658*; *SLC26A4*; *LOC121819399*	ROH + FST + XP‐EHH
	18	60.03–60.78	*LOC114109133*	iHS + FST + XP‐EHH
	2	99.88–101.75	*LOC105608475*; *LOC105608482*; *LOC121818666*; *LOC121818667*; *ACO1*; *TRNAG‐UCC*; *DDX58*; *TOPORS*; *SMIM27*; *NDUFB6*; *TRNAS‐GGA*	ROH + FST
	6	62.08–64.53	*ATP8A1*; *GRXCR1*; *KCTD8*; *YIPF7*; *GUF1*; *GNPDA2*; *LOC114115307*; *TRNAW‐CCA*	ROH + XP‐EHH
	22	33.62–35.38	*LOC101122236*; *CASP7*; *TRNAG‐CCC*; *PLEKHS1*; *DCLRE1A*; *NHLRC2*; *LOC105604300*; *ADRB1*; *CCDC186*; *TDRD1*; *TRNAC‐GCA*; *VWA2*; *AFAP1L2*; *TRNAY‐GUA*; *ABLIM1*; *FHIP2A*; *TRUB1*; *ATRNL1*; *TRNAC‐ACA*	ROH + XP‐EHH
	1	155.96–157.84	*VGLL3*; *LOC121816279*; *CHMP2B*; *POU1F1*; *HTR1F*; *LOC105611303*; *CGGBP1*; *ZNF654*; *C1H3orf38*; *LOC105616630*	iHS + XP‐EHH
Batur	6	22.73–23.23	*SLC39A8*; *BANK1*; *LOC114115496*	iHS + XP‐EHH
	2	86.65–87.46	*ADAMTSL1*; *LOC114113270*	iHS + XP‐EHH
Sakub	2	162.53–163.53	*TRNAS‐GGA*	iHS + XP‐EHH
	9	20.34–21.43	*ZFAT*; *LOC105608712*; *ST3GAL1*; *LOC121820317*; *NDRG1*; *TRNAC‐ACA*; *CCN4*; *LOC121820382*; *TG*; *SLA*	iHS + XP‐EHH
Thin tail	5	14.26–14.96	*FCER2*; *LOC114114996*; *LOC101123627*; *LOC101102057*; *EVI5L*; *PRR36*; *LRRC8E*; *MAP2K7*; *TGFBR3L*; *SNAPC2*; *CTXN1*; *TIMM44*; *ELAVL1*; *CCL25*; *LOC121819602*; *FBN3*; *TRNAR‐CCU*; *CERS4*; *CD320*; *NDUFA7*; *RPS28*; *KANK3*; *LOC121819603*; *ANGPTL4*; *RAB11B*; *MARCHF2; HNRNPM*; *PRAM1*; *ZNF414*; *LOC121819605*; *MYO1F*; *LOC121819604*; *ADAMTS10*; *NFILZ*	iHS + XP‐EHH
	13	56.70–57.20	*EDN3*; *ZNF831; PRELID3B*; *ATP5F1E*; *TUBB1*; *LOC121816190*; *CTSZ*; *NELFCD*; *LOC101115640*; *LOC101102411*; *LOC121816191*	iHS + XP‐EHH

The most robust putative regions were detected in *Sumatra* sheep on chromosomes 3, 4 and 18, while secondary putative selection regions were identified on chromosomes 1, 2, 6 and 22. Putative positive selection signature genes detected here have been reported in multiple traits in sheep, e.g., in reproduction traits; *GNPDA2* genes that play roles in metabolic transport have effects on rams' sperm capacitation process (Chen et al. 2024), *NAMPT* in ovarian function during luteal phase (Li et al. 2023), *ABLIM1* with fecundity (Miao et al. 2018) and *POU1F1* with pituitary function (Li et al. 2025). For growth traits, there were *PIK3CG* with muscle development in Tibetan sheep (Liu et al. 2025), *TRNAS‐GGA* with body weight in Merino sheep (Krivoruchko et al. 2020), *CDHR3* with cell adhesion in lowland Tibetan sheep (Tian et al. 2023), *ACO1* with lipid metabolism (Yu et al. 2026) and *VGLL3* with muscle fiber composition (Liu et al. 2025). While for adaptation, *SLC26A4* in thermal adaptation and fluid balance (Pang et al. 2025), *NDUFB6* in metabolic adaptation (Gu et al. 2025), *ATRNL1, DDX58* and *ADRB1* in immune response (Qi et al. 2025; Pinto et al. 2025; Arzik et al. 2026) and *CASP7* in apoptosis (Lu et al. 2022), have been reported in sheep studies.

For *Batur* sheep, secondary putative positive regions were located in chr 2 and 6, covering the gene locations of *SLC39A8, BANK1, LOC114115496, ADAMTSL1*, and *LOC114113270*. *SLC39A8* is linked to mammary gland expansion during the lactation period (Chen et al. 2022), *ADAMTSL1* is reported to affect lambing interval (Abdoli et al. 2019), while *BANK1* is linked to feed efficiency (Zeng et al. 2023). At the nominal significance level, GO BP extracellular matrix organization (GO:0030198) was enriched in Batur sheep; however, this finding should be considered exploratory because no GO terms remained significant after multiple‐testing correction.

Chromosomes 2 and 9 in *Sakub* breed were determined to be putative positive selection regions, compromising 11 genes. Within this region, *ZFAT*, a placental‐expressed gene linked to female reproduction (Gong et al. 2023), *ST3GAL1*, a milk protein‐related gene (Rezvannejad et al. 2022), *NDRG1*, a spermatogenesis, cell growth, and differentiation‐related gene (Qu et al. 2019), and *CCN4*, a wound healing‐related gene in goat (Zheng et al. 2023), were detected.

As much as 45 genes were detected as putative positive selection signatures for *Thin‐tail* sheep, which were located in chr 5 and 13. Some of these genes have been reported previously to be linked to ovine production traits: *FCER2* and *NELFCD* to immunological responses (Yaro et al. 2019; Zhao, Mu, et al. 2024), *EVI5L* to milk synthesis (Marei et al. 2025), *CERS4* to hair follicle development and wool quality (Wang et al. 2025), *EDN3* to hyperpigmentation (Darwish et al. 2018), *NDUFA7* to oxidative phosphorylation process of lamb oocytes (Ying et al. 2021), *ANGPTL4* to fat deposition (Fu et al. 2026), and *ZNF381*, *CTSZ* and *PRELID3B* linked to early lactation activation and milk production (Zhao, Mu, et al. 2024; Akhatayeva et al. 2026).

Multimethod detection of putative selection signatures was employed in this study to improve robustness; however, the functional interpretation of candidate genes identified should be considered preliminary, as associations are only based on positional overlap and evidence from other species. Therefore, further validation using phenotypic, functional, or environmental data is highly required. Nevertheless, these findings provide a preliminary genomic reference for future breed‐specific improvement and conservation efforts in Indonesian sheep.

## 5 Conclusions

This study provides the first medium‐density SNP‐based genomic characterization of five local Indonesian sheep breeds. Population‐structure analyses indicated distinct ancestry patterns for Batur, Garut, and Sumatra, while Sakub and Thin‐tail showed close genetic relatedness. Using complementary within‐ and between‐population approaches, we identified 13 putative selection‐signature regions containing genes previously reported in association with reproduction, production, immunity, and adaptation‐related traits in sheep. Given the reduced genomic resolution after dataset harmonization, these findings should be considered preliminary and require validation using denser genomic and phenotypic data. The relatively elevated genomic inbreeding observed in some breeds also supports the need for continued monitoring in future breeding and conservation programs.

## Author Contributions

**Putri Kusuma Astuti:** conceptualization (equal), data curation (equal), formal analysis (equal), methodology (equal), visualization (equal), writing – original draft (equal). **Alexandru Eugeniu Mizeranschi:** data curation (equal), formal analysis (equal), visualization (equal). **Herdis Herdis:** methodology (equal), writing – review and editing (equal). **Pradita Iustitia Sitaresmi:** methodology (equal), writing – review and editing (equal). **Alek Ibrahim:** methodology (equal), writing – review and editing (equal). **Sigit Prastowo:** methodology (equal), writing – review and editing (equal). **Nuzul Widyas:** methodology (equal), writing – review and editing (equal). **Tristianto Nugroho:** methodology (equal), writing – review and editing (equal). **Szilvia Kusza:** conceptualization (equal), funding acquisition (equal), supervision (equal), writing – review and editing (equal).

## Ethics Statement

The Local Ethics Commission of Universitas Sebelas Maret, Indonesia, approved the sampling for this study (Registry number 041/EC/05/25). All experiments were conducted in compliance with national legislation on animal research and the ARRIVE guidelines.

## Conflicts of Interest

The authors declare no conflicts of interest.

## Supporting information


**Table S1:** PCA.
**Table S2:** Admixture.
**Table S3:** ROH analysis.
**Table S4:** SS‐ROH.
**Table S5:** SS‐iHS.
**Table S6:** SS‐FST.
**Table S7:** SS‐XP EHH.
**Table S8:** SS‐Final regions.
**Table S9:** Annotated genes.
**Table S10:** GO & KEGG.

## Data Availability

The data generated from this study are publicly accessible via DOI https://doi.org/10.5281/zenodo.18210214. The Supporting Information is available together with this manuscript.
